# Cultivating Cooperative Relationships: Identifying Learning Gaps When Teaching Students Systems Thinking Biomimicry

**DOI:** 10.3390/biomimetics7040184

**Published:** 2022-10-31

**Authors:** Laura Lee Stevens, Celina Whitehead, Asha Singhal

**Affiliations:** 1 Industrial Design Engineering, The Hague University of Applied Sciences, 2521 EN Den Haag, The Netherlands; 2 Hybrid Futures, Strassmannstr., 10249 Berlin, Germany

**Keywords:** biomimicry, systems thinking, pedagogy, design thinking, science education, biology

## Abstract

The methodology of biomimicry design thinking is based on and builds upon the overarching patterns that all life abides by. “Cultivating cooperative relationships” within an ecosystem is one such pattern we as humans can learn from to nurture our own mutualistic and symbiotic relationships. While form and process translations from biology to design have proven accessible by students learning biomimicry, the realm of translating biological functions in a systematic approach has proven to be more difficult. This study examines how higher education students can approach the gap that many companies in transition are struggling with today; that of thinking within the closed loops of their own ecosystem, to do good without damaging the system itself. Design students should be able to assess and advise on product design choices within such systems after graduation. We know when tackling a design challenge, teams have difficulties sifting through the mass of information they encounter, and many obstacles are encountered by students and their professional clients when trying to implement systems thinking into their design process. While biomimicry offers guidelines and methodology, there is insufficient research on complex, systems-level problem solving that systems thinking biomimicry requires. This study looks at factors found in course exercises, through student surveys and interviews that helped (novice) professionals initiate systems thinking methods as part of their strategy. The steps found in this research show characteristics from student responses and matching educational steps which enabled them to develop their own approach to challenges in a systems thinking manner. Experiences from the 2022 cohort of the semester “Design with Nature” within the Industrial Design Engineering program at The Hague University of Applied Sciences in the Netherlands have shown that the mixing and matching of connected biological design strategies to understand integrating functions and relationships within a human system is a promising first step.

## 1. Introduction

Worldwide, there are increasing concerns over the effects of climate change (Ogunbode et al., 2020 [1] including environmental pollution, loss of biodiversity, extreme weather events and more. With the rising climate crisis, a new movement known as an “eco-awakening” (WWF, 2021) has led to an interest in looking to nature for more circular and sustainable alternatives. Nature’s emphasis on providing closed-loop and regenerative systems has created a shift towards the exploration of ecology in design. Many pedagogical disciplines, such as ecologically sustainable design (ESD), regenerative design, ecomimicry and biomimicry aim to understand the methods of bio-integration to advocate natural solutions to complex human challenges (Benyus, 1997; Bhushan, 2009; Mochizuki and Fadeeva, 2010; Dicks, 2015; Dicks and Blok, 2019) [2,3,4,5,6]. Responsible innovation and biomimicry are earning recognition as businesses strive to implement systems thinking biomimicry as part of their strategy in projects. Faludi and Gilbert [7] mention systems thinking, communication and interdisciplinarity within teams as aspects to be considered “best practices”. In fields such as the built environment, fashion, and industrial design, there is a need to implement systemic approaches to the design process which not only lessen damage but instead provide positive impacts of restoration, renewal and regeneration (Zari, 2018) [8].

Benyus (1997) [2] describes biomimicry as imitating or taking inspiration from nature’s forms, processes and ecosystems to solve problems for humans. Satori, Pal and Chakrabarti et al. (2005) [9] describe the SAPPhIRE model used for biomimetic design to identify similarities in biological and corresponding analogical transfer to technical systems. Yen, Helms, Goel, Tovey and Weissburg described their university curriculum for biologically inspired design (BID) and its potential in interdisciplinary education where existing knowledge from nature could be applied into a new field of engineering design (Yen et al. 2010, Yen et al., 2011, Yen et al., 2014) [10,11,12]. Early function-based bio-inspiration and concept generation, such as what Nagel and Stone (2011) [13] introduced, guided engineering students through an abstraction approach. With this approach, students used inventive problem-solving tools, TRIZ4 (Vincent et al., 2006) [14] and BioTriz (Bogatyrev and Bogatyreva, 2014) [15], before translating the biological systems into systemic reverse engineering. Vincent et al. (2006) [14], Yen and Weissburg (2007) [16] and Goel, Vattam, Wiltgen and Helms (2014) [17] also describe the translation of mechanisms being function-based and not necessarily form-based. Highlighting this critical distinction requires acknowledgment of mimicking functions, as replicating form may not be necessary or perhaps impossible. Pawlyn (2016) [18] describes the field as design that is inspired through biology’s manner of solving functional challenges. El-Zeiny (2012) [19] shares this meaning, emphasizing against understanding biomimicry as a shape-copying design practice. The discipline of biomimicry has been described as a “unique problem-solving approach, linking science and art in a methodology, a technology, a process and an ethic” (Rovalo et al., 2020) [20].

Educational institutions add biomimicry to curricula while developing environmental education and skills such as systems thinking (Qureshi, 2020; De Pauw, 2015) [21,22]. For the purpose of this paper, we will mainly focus on systems-level emulation methodologies in biomimicry which reiterate the understanding of multifunctional strategies within a natural system, in order to be able to implement these strategies in practice. Zari (2010) [23], Kennedy et al. (2015) [24] and Baumeister (2014) [25] describe the field as the translation of an organism’s form or structure, its behavior or an entire ecosystem in terms of how these support the function. Baumeister mentions that if we are able to mimic all three—form, process and ecosystem—we will start learning how to adapt to and create conditions “conducive to life”. This mimicking of form, process and ecosystem with a focus on the function these perform are the three levels of emulation within biomimicry design, and address the essential element of Biomimicry 3.8′s “Emulate” or create (Benyus, 1997; Baumeister, 2014) [2,25]. Biomimicry 3.8, AskNature.org and The Biomimicry Institute emphasize that this is only one of three essential elements, where “Ethos” (ethical design decisions) and “Reconnect(ing to nature)” are the other two. Without considering all three, an essential sustainability benefit could be missing. It is this definition and practice that is used during this study, as the authors have found all three to be essential to how students learn. Definitions of biomimicry outside of this boundary were considered; however, they are outside the scope of this study. This study describes how these three “essential elements” steer the focus of this article and the learning path of students learning biomimicry.

Biomimicry design education is emerging rapidly as The Biomimicry Institute and graduates from biomimicry higher education programs continue to replicate the learned knowledge and branch out. Higher education biomimicry programs are provided by universities such as Arizona State University (ASU), Oregon State University, Akron University and University of California in USA, The Hague University of Applied Sciences, Utrecht University, Karadeniz Technical University in Turkey and MSA University in Cairo, Egypt, to mention a few. Universal pedagogical challenges such as translating the literal mechanism into the product solution are only slowly being overcome. The ability to apply knowledge from one context, such as biology, into a different context, such as design, where similarities and remote associations are found, is called analogical reasoning (Kolodner, 1997) [26]. Vattam, Wiltgen, Helms, Goel and Yen introduced a library of engineering design indexed by their functions, called “The Design by Analogy to Nature Engine” or DANE (2011) [27].

When we use the terms form, process and system analogies in this paper, we refer to such an application and transfer of these kinds of functional strategies and mechanisms as “levels” of biomimicry analogies. Form-level biomimicry focuses on mimicking shapes and structures in nature, while process-level biomimicry focuses on emulating behaviors and manufacturing processes. Systems-level biomimicry, however, focuses on a deeper understanding of the interactions between elements in nature and emulation of the larger system (Baumeister, 2014) [25]. System-level biomimicry places an emphasis on designing closed-loop, multifunctional, systemic and regenerative solutions learnt from nature’s overarching patterns and processes (Baumeister, 2014) [25]. Jones and Kijima (2018) [28] describe systemic design as an approach useful for solving complex problems in a holistic manner while using design reasoning and scientific principles. Systems theory provides a method to incorporate and organize patterns. Systems thinking biomimicry brings the concept of thinking in systems together with design thinking and biology to solve design challenges while focusing on systemic sustainability (Rowland, 2017) [29]. The biological (bio)mimicry levels of form (structure) and process (behavior) are more easily seen and understood by students, while systems-level biomimicry remains complex (Shu and Cheong, 2014; Stevens, 2021) [30,31].

Yeler (2015) [10] shares the importance of student comprehension of how biological strategies in nature can be translated to design solutions and emphasizes how integration of such teachings into education is a priority to help create a sustainable world. “Consideration of the overall, although design teaching has occasionally looked at nature for inspiration, systematic approaches to teaching design fundamentals through nature have been rare” (Yeler, 2015, p. 408) [32].

While biomimicry offers guidelines and methodology, there is insufficient research on complex problem solving that systems thinking requires in biomimicry practice. The research question asks which factors are needed to help (novice) professionals initiate systems thinking methods as part of their strategy. A solution should enable them to approach challenges in a systems thinking manner just like nature does, to regenerate and resume projects. With this effort, together with the students we look to define elements which helped them understand how systems have relationships and multiple connections in biomimicry education. The found elements are starting points to expand on in future research studies. Biomimicry as a practice specifically aims to contribute to sustainable solutions that are ethical and that help reconnect humans with nature (MacKinnon et al., 2020) [33]. Conscious and scientifically correct emulation of life’s genius is key to this. Baumeister describes the importance of requiring specific intent and research to mimic strategies from adaptations that have endured the test of time, and subsequently applying these strategies to human challenges in need of that same function (2014) [25].

### Building from the Bottom Up: Past Research on Overarching Patterns in Nature

Life’s Principles are a set of patterns exhibited by nature that contribute to life’s ability to survive and thrive. These patterns serve as both guidelines and benchmarks for biomimicry thinking design phases (Baumeister, 2014) and organized into six main categories by the Biomimicry Guild, now Biomimicry 3.8, in the year 2000 (Biomimicry 3.8, 2015) [34]. These patterns need guidelines, which many biomimics (practitioners of biomimicry) find in biomimicry’s Life’s Principles. According to Patel and Mehta (2011) [35], these principles are described as the building blocks in nature, leveraging interdependence within a continually optimizing system. Nature integrates these principles, such as multi-functionality, using readily available materials and energy, instead of producing and shipping parts across the globe, and all the consequences thereof.

**Figure 1:**
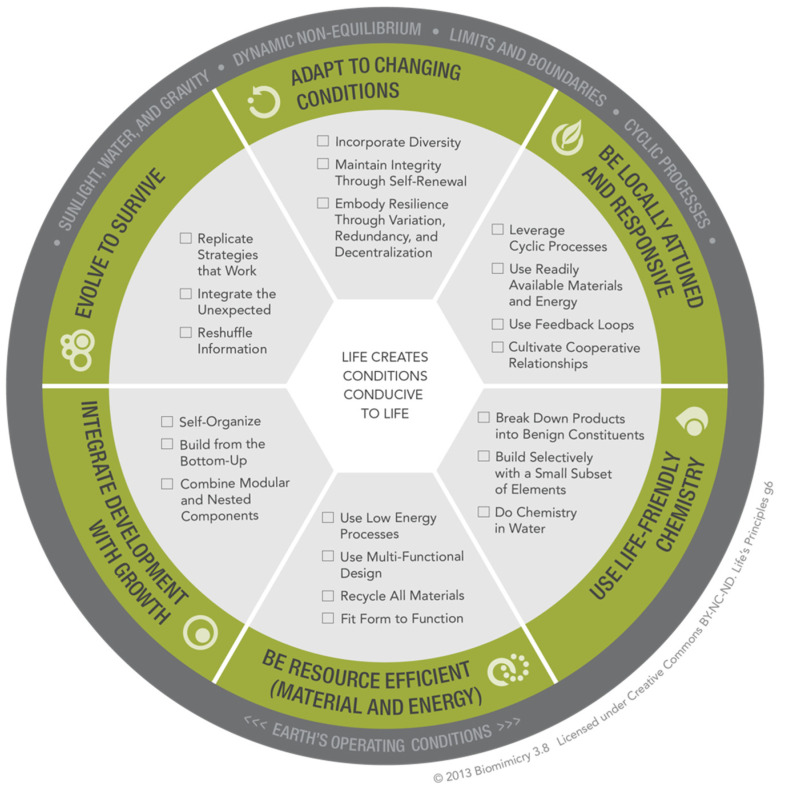
Six main biomimicry Life’s Principles and their sub-principles. ©2015 Biomimicry 3.8. CC BY-NC-ND. Permission granted by Biomimicry 3.8 under Creative Commons [34].

The set of Life’s Principles (LPs) can serve as a resource within the biomimicry methodology for qualitative goal setting in the scoping phase and for holistic system-level design analysis during the evaluation phases (Figure 1). Life’s Principles (LPs) provide us a summary of the deep patterns found in natural systems that help create conditions conducive to more life or, in other words, patterns that can serve as metrics for the success of sustainability. If these are considered and applied in design solutions, this also demonstrates the ethical effort to meet the sustainability requirements (Kennedy et al. 2015) [24]. The authors acknowledge the value of integrating biomimicry’s LPs for enhancing systems thinking. For example, “Use Feedback Loops” is one of the sub-principles under “Be locally attuned and responsive”. Feedback loops refer to the set of signals and responses that nature uses to thrive. An example of a human design using feedback loops might be how a thermostat registers the temperature and signals the heater to turn on/off. The set of LPs is an essential tool to be integrated into the design process during each phase and is described in detail in the *Biomimicry Resource Handbook* (Baumeister, 2014) [25] and *Building from the Bottom Up: A Closer Look into the Teaching and Learning of Life’s Principles in Biomimicry Design Thinking Courses* by Stevens, Fehler, Bidwell, Singhal and Baumeister (2022a) [36]. However, with this study, we investigate *additional* requirements that a professional systems thinking biomimicry “tool-kit” or roadmap needs, as Life’s Principles have already been firmly established within our own education through this earlier research. One option to do so is the use of systems thinking biomimicry. The authors describe this thinking as applying a combination of systems thinking and biomimicry design thinking to holistically approach a design challenge.

Previous manuscripts with the leading author (2021; 2022a) [31,36] describe how difficult it is to implement technical requirements while practicing biomimicry. Nagel et al. (2010) [37], Vattam, Helms and Goel (2008) [38]; Vattam, Wiltgen, Helms, Goel and Yen (2011) [27] and Rovalo et al. (2020) [20] depict the translation between the biological design principle (BDP) and the Abstract Design Principle (ADP) as a “fundamental hurdle”. Nguyen and Bosch (2013) [39] explain the importance of considering the system and identifying its interconnected parts for a framework to manage change within complex situations and to interpret the consequences of intervention. A key aspect of systems thinking relies on causal loops focusing on cause and effect in the form of feedback loops. As proposed by Meadows in her book *Thinking in Systems*, knowing the leverage points within that system is thus essential to understanding where and how to intervene while also solving the issues that arise due to the effects of each design decision. Leverage points refer to where a designer might intervene between connected links, relationships and other issues in a system (Meadows, 1997) [40].

In this manuscript, the authors address the issue our future professionals’ need to face, in particular, the difficulty for designers to start adopting biomimicry into their professional practices. Clients involved in the past cohorts of Design with Nature (DwN) at The Hague University of Applied Sciences (THUAS), have repeatedly stated the need to apply systems thinking to design challenges. They have emphasized a need to understand how the larger system of the design challenge can function optimally and where they might intervene within these systems to make an impactful change. In addition, when a change is made, how might this affect the balance of the system itself without making things worse. The objective of this research is to discover which factors aid professionals-to-be to develop, initiate and continue systems thinking biomimicry projects as part of their design strategy. Our starting point for this article is to view students and their perception of what has helped them during their learning of biomimicry and systems at The Hague University of Applied Sciences.

## 2. Materials and Methods

A major element in this shift towards nature-inspired design is fulfilling the needs of professionals beyond their initial enthusiasm so they feel enabled to make a holistic positive impact without unintended adverse consequences. To address this challenge, methods from the field of systems thinking were incorporated with the biomimicry methodology to work with systems thinking biomimicry. In this study, the criteria for determining if students were successful in thinking in systems was less important than what students *felt* helped them move further with their design. We explained that systems thinking needed to consider multiple connections and relationships within the collaborative challenge each team was trying to solve for their stakeholder (within the system of stakeholders or clients involved in the semester). Students were challenged to look at how a solution might have an effect on the system it is involved with. “Many of the human interactions/behaviors/structures in current societal systems and subsystems (e.g., education, health, finance, politics, etc.) are considered by many to be ‘broken’, despite the best intentions of their original designers. Built on deep patterns of fear, scarcity and competition, they are in need of redesign and transformation” (Prakash et al., 2019. p. 3) [41]. This study aims to guide students to finding their own “best practices” to approach biomimicry challenges in a systematic manner.

The overarching research question is:

Which factors are needed to help professionals initiate, develop and continue systems thinking biomimicry projects as part of their design strategy?

Our sub-questions are:

- RQ 1: Which elements of biomimicry methodology supported both designers and non-designers within a team to continue and increase their interest in learning and which factors were relevant for specifically form, process and system analogies?
- RQ 2: According to participating students, which exercises within the phases of biomimicry design thinking assisted their attempts to apply systems thinking?
- RQ 3: What pedagogical or didactical factors in biomimicry methodology did students struggle with or find missing?

To answer the research questions, a mixed-model approach was used to improve result validity (Khakpour, 2012) [42]. In phase 1, the student survey took place at the end of second week of the semester after the main biomimicry workshops of the following tools: systems mapping, BioBrainstorming, Life’s Principles, biological design principle (BDP), abstract design principle (ADP) and Nature Technology Summary (NTS). These tools will each be explained in more detail in the following sections (except Life’s Principles). In phase 2, interviews of the 2022 cohort aimed to verify the findings in order to designate design requirements for a new biomimicry education tool focused on systems. In the semi-structured interviews, questions are prepared beforehand but aim for flexibility to build on student responses. This flexibility allowed the authors to navigate the interview direction to determine how the research would benefit both the instruction of biomimicry design and the participants in the semesters (Rowell et al., 2015) [43]. This qualitative research method had the advantage that the interviewer could be flexible to observe and build upon the reactions of students while gathering information that would otherwise not have been possible. A disadvantage was the relatively high cost of time investment for the lead researcher.

Surveys and interviews also ensure a rapid turnaround in data collection or, rather, feedback loops. Semi-structured discussions and interviews aim to hone in on detailed information (Driscoll, 2011; Creswell, 2014) [44,45]. The survey responses function as quick feedback loops to ensure that the researchers were able to adapt to the students’ learning patterns directly in following sessions. In these sessions, all students were continually encouraged to go beyond form and behavior and to look for relationships and (eco)systems. We collected and reviewed the responses of students to cluster and restate what students said while highlighting patterns. Assignments are described in the sections below and “coding” texts in both surveys and interviews are clustered according to key words such as “connected” and “map out relationships” as being related to systems.

It is important to note that our questions to participants aim to find out what students themselves designate as helpful to their own learning process. It is the student perception that we measure to provide results for pedagogical guidelines for future courses. Finding out what students feel helped them in systems thinking serves as a catalyst for their own future design processes. By honing in on student perception, we aim to support their self-driven learning process. Through our surveys and interviews, our aim is to narrow the “transactional distance” (Saba, 2007) [46] of learners which is related to their perceived learning. Mullen and Tallent-Runnels (2006) [47] describe this support as positively relating to student learning outcomes, achievement and satisfaction of the course. This aids student motivation to learn (McCombs and Marzano, 1990) [48]. Saba (2016, p. 22) [49] also emphasizes that a consistent effort should be taken to “place the learner at the center of the teaching-learning process, with more active and responsible roles”.

Student population (Table 1) is undergraduate levels, ranging across a variety of disciplines. The disciplines of students included but were not limited to design, communication, physics, mechanical engineering, built environment and urban studies. Extra effort is taken to inform participants of this research, to involve them in each phase and to ensure a safe environment for all concerned.

### Exercises in This Study

The workshops given before the survey were focused on the following biomimicry pedagogical elements: (1) mapping out aspects within a challenge and how each affects others within a system (system mapping); (2) investigating multiple biological strategies solving a specific function (BioBrainstorm); (3) drawing and specification of the biological design principle (BDP) strategy responsible for this function; (4) abstracting these strategies into coherent design principles (ADPs) including respective diagrams which explain the strategy clearly, both textually and visually, without the biological terms; (5) The detailed Nature Technology Summary (the collection of all found information on one organism and one function); and (6) the integration of Life’s Principles characteristics into the design. This last exercise is not included in the data collection, as it has been proved to be a successful instrument for systems thinking in earlier biomimicry research.

The five exercises and matching tools that are explained in this section (plus Life’s Principles) were given to students during the first unit of the semester:

- Systems-mapping;
- Bio-Brainstorm;
- Biological design principle;
- Abstract design principle;
- Nature Technology Summary.

**Figure 2:**
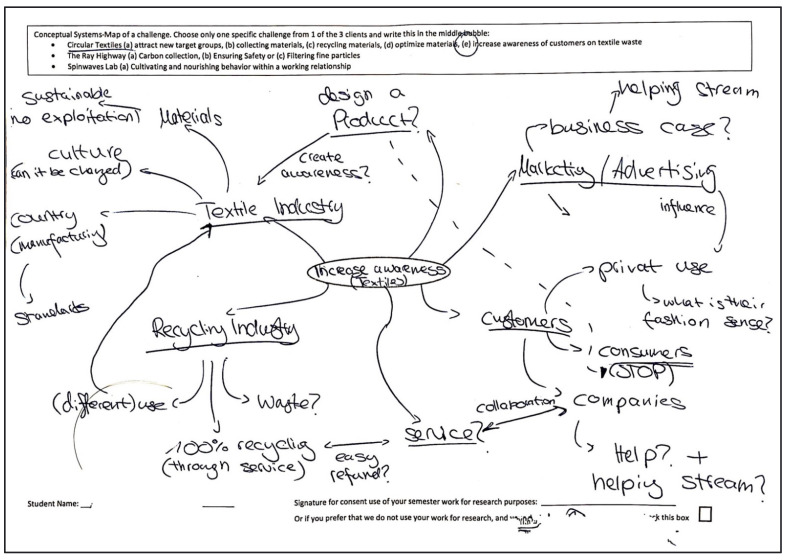
Example of a systems-mapping exercise.

System mapping (or concept mapping) is an activity in which the visual aspect is useful to translate abstract knowledge of students’ learning into concrete visual representations that can be compared without disruption to conventional teaching methodology (Hay et al., 2008) [50]. At the start of each semester, students were asked to draw a “systems-map” of one of the three main challenges to be addressed during the semester. For example, in 2022, students could choose from the following challenges: (1) circular textiles from production, collection and retail to recycling, business and designer clothing, (2) roadside CO_2_ sequestration, safety and filtration of particles, and (3) how might stakeholders cultivate cooperative relationships within working relationships. The 20 min exercise instructed students to add as many aspects of the challenge that they could think of, to connect relationships and to mark directional flows of resources. After this, they were asked to discuss their maps with other students to determine where they might be able to intervene, and which of these interventions might lead to having the highest positive impact (Figure 2).

**Figure 3:**
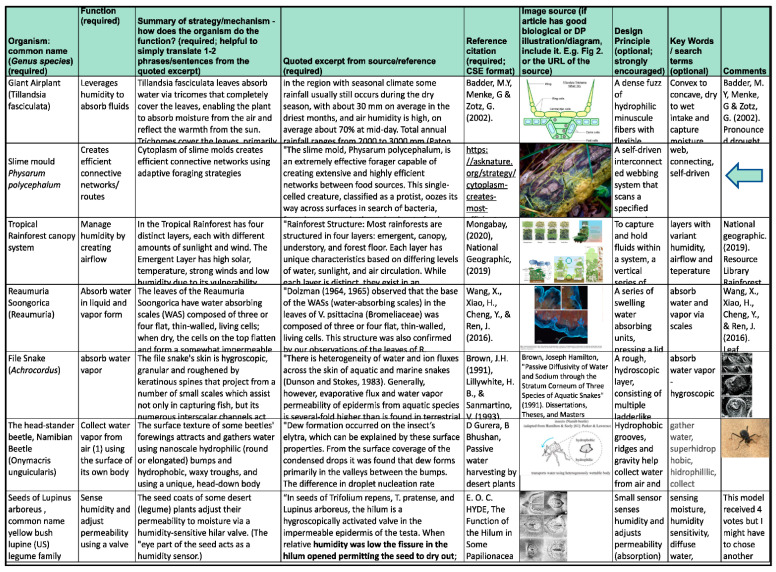
Example of BioBrainstorming and research.

A BioBrainstorm is a biomimicry research activity to register multiple organisms and natural systems sharing one successful function. It consists of recording the name of the natural organism/system, the strategy for that function, the mechanism describing how the strategy works and the (abstracted) design principle. A tool that can be used to keep track of the different organisms, functions, etc., is an online worksheet (Figure 3). This worksheet enables teammates within a specific challenge to see which organisms and respective mechanisms have already been found in primary research documents and registered in their ongoing BioBrainstorm list. Students were asked to add six unique organisms to their teams’ BioBrainstorm sheet within the period of a week.

**Figure 4:**
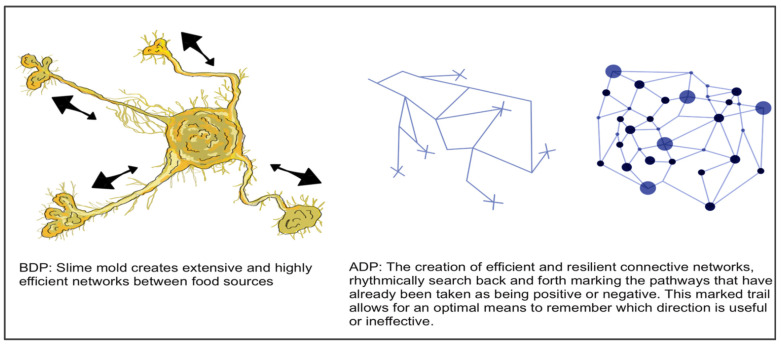
BDP and ADP diagram of slime mold—I. Rössler and C.Whitehead, 2021 cohort.

A biological design principle (BDP) is a deep principle derived from nature, focusing on one specific function an organism/natural system performs well, including a descriptive text and corresponding diagram (Figure 4, left). An abstracted design principle (ADP) is a deep principle from nature stated in non-biological terms, including a descriptive text with a corresponding diagram (Figure 4, right). Students were asked to describe and hand draw each of their found organisms as homework before the following lesson 2 days later.

**Figure 5:**
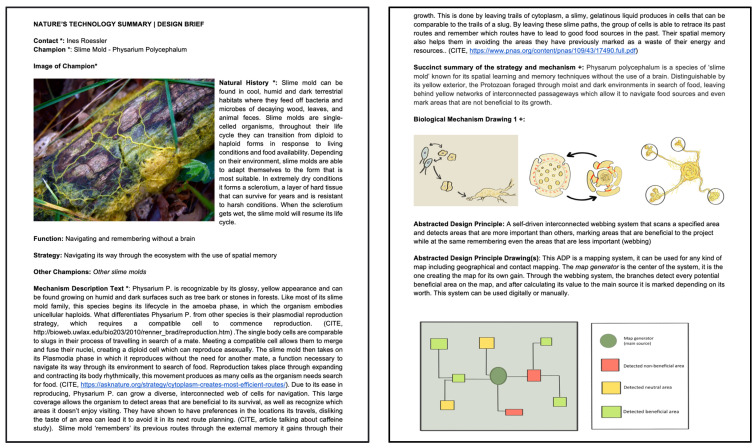
Example of a Nature Technology Summary (NTS). I. Rössler and C.Whitehead, 2021.

A Nature Technology Summary (NTS) is a template for summarizing the research from primary resources and abstracted design principles (ADP) for biological mentors. It contains scientific and common names, function, natural history, strategy, mechanism, biological design principle (BDP) and ADP, including diagrams, Life’s Principles (LPs) and references (Figure 5). Students were asked to complete at least one NTS before the survey at the end of the next lesson. We calculated and advised students that just one NTS may take 15–20 h of work. Each section of the template was practised for one organism in class.

Previous cohort work has been reviewed to find successful examples of systems- level biomimicry. A potential indication of success is the team’s own perceived ability to link relationships and functions within a natural ecosystem to those within the client’s design challenge. We could choose to score final product solutions as to how these setup such relationships and related functions. However, with this first step we choose to gauge student engagement and perception instead, which served as the main indicator of success for the purposes of this study.

In phase 1 of this study, 25 students (face-to-face cohort DwN2022) responded in a survey about their design steps. We recorded elements of the lessons that students mention as being helpful to their understanding as to what was responsible for this. The open questions in the survey functioned both as a starting point to (1) gauge the level of this analogical transfer and (2) discover which pedagogical elements were interesting or helpful for the students who were successful in recognizing systems analogies between biology and design ideation. Bradford et al. (2016, p. 36) [51], describes how “a more self-defined mastery of material from the student perspective led to greater achievement”. Thus, asking students to make a statement on their own interests in learning should improve retention, e.g., (Nichols, 2010) [52]. The survey also required students to relate success in mimicking form, process or system in a workshop, to the exercise helping to achieve this level. We focused on “systems” responses for this phase. However, responses from all levels were recorded to find relationships between student understanding and the degree of perceived success of specific methods or exercises they used. Through these surveys, we also aim to gather first assumptions on which elements have remained clear and which elements have remained uninternalized.

In phase 2, semi-structured interviews on systems thinking aim to verify initial findings by speaking in depth with 10 of the same students from the survey. The researchers recognize that the voluntary manner in which students participated in the interviews may affect responses. Questions are prepared beforehand but are purposely flexible to build on student responses (Drever, 1995) [53].

## 3. Results

### 3.1. Phase 1 Survey Students’ 2022 Cohort

In phase 1, a survey for the Design with Nature 2022 cohort aimed to gather insights. Here, 25 students responded to the survey after learning how to make the analogical leap to practice the translation between biological and design strategies. The biomimicry methods taught before the survey were BioBrainstorming, Life’s Principles, biological design principles and abstracting these principles, as well as complete Nature Technology Summaries (NTSs) of found organisms. More students than ever before in Design with Nature felt they were mimicking (eco)systems. The students were split between drawing activities and writing activities when asked about what cultivated their interest. A smaller number of students mentioned the biology lessons. Most students felt that learning the translation from biology to design was difficult but rewarding.

**Figure 6:**
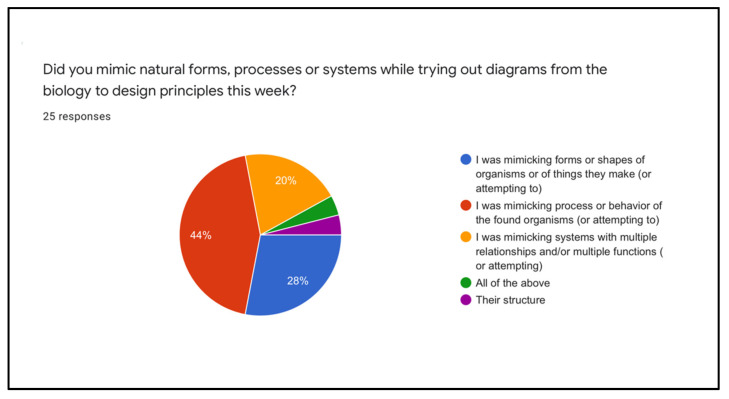
Student responses to analogy type. (small section pie 4% each).

We asked students whether they felt they were successful in mimicking form, process or system, and they had the choice of each of the three plus “*all of the above*” and “*other*” (Figure 6). With 32% mimicking form (including 4% who felt that they were successful in mimicking structure), 44% mimicking process and 20% mimicking system, these numbers show a higher level of systems analogies than any cohort taught between 2019 (Stevens, 2021) and 2022 [31,36,54,55]. One student recalled mimicking all three levels.

**Figure 7:**
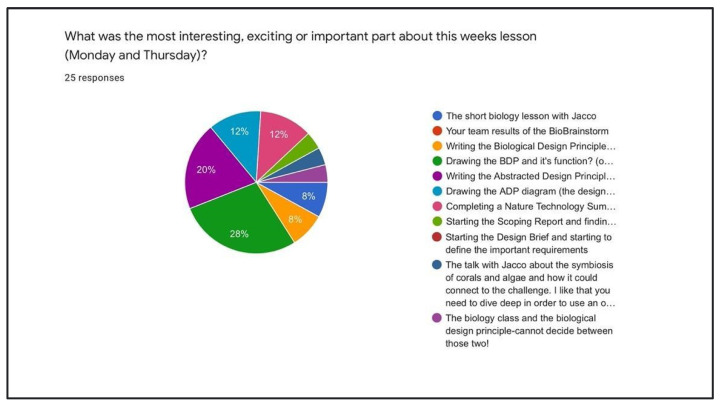
Student cohort of 2022 responses to “most important” exercise. (small section pie 4% each).

When prompted to respond to what was exciting or interesting in the classes using a single-choice survey, 40% (10 students) chose an action concerning drawing by hand, such as “drawing the Biological Design Principle (BDP)” or “Drawing the Abstracted Design Principle (ADP)”. Another 40% chose the actions involving research or interpreting the found biological research into written summaries connected to the function–need they were challenged with. The responses “Writing the Biological Design Principle (BDP)”, “Writing the Abstracted Design Principle (ADP)”, “Completing a Nature Technology Summary (NTS)” or “Starting the Design Brief” were marked in the research category. Another 20% chose other activities related to lessons where they were given information, such as a biology lecture or a lesson on scoping the challenge (Figure 7).

**Figure 8:**
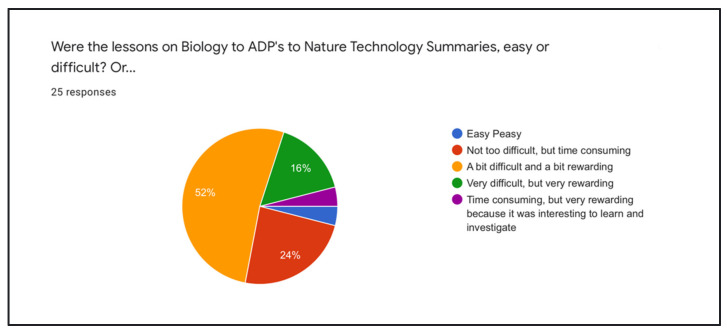
Student cohort of 2022 responses to ease of translation exercises. (small section pie 4% each).

Students also responded about how difficult or how rewarding the BDP, ADP or NTS exercises were. A total of 52% responded “A bit difficult and a bit rewarding” and 20% responded “Very difficult/time consuming, but very rewarding”. This question was on a scale from “Easy Peasy” to “Very Difficult, but very rewarding” or “Other” (Figure 8). These questions were asked during the first unit, in which all of the biomimicry tools were introduced for the first time. In phase 3, the students are asked specifically about these three exercises and how difficult/rewarding they turned out to be at the end of the semester.

To correlate the responses from the 2022 cohort with what we learned from earlier research, we grouped the students’ responses to the survey into separate categories for the types of analogical reasoning (form, process and system—the student who responded to all three was added to the systems sheet). Each group was then color-coded into categories to provide insights into overarching patterns.

**Figure 9:**
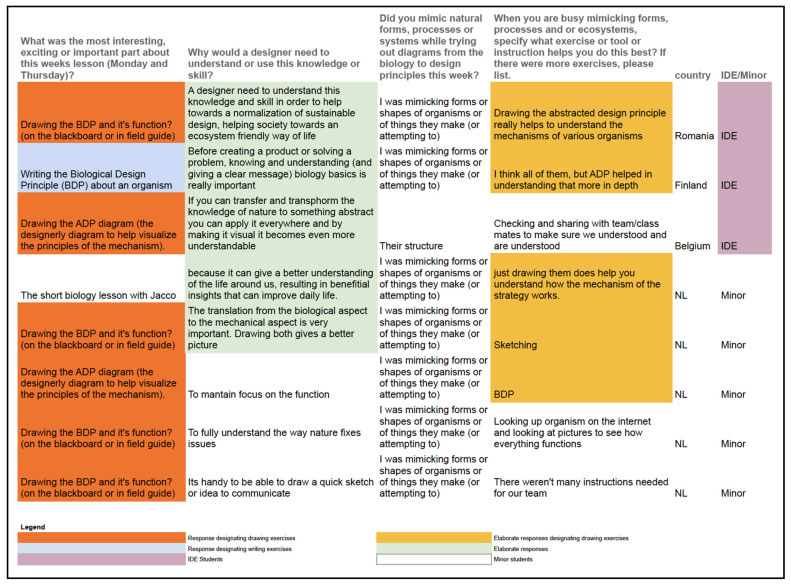
Interview responses of the student cohort mimicking *form* in their designs.

**Figure 10:**
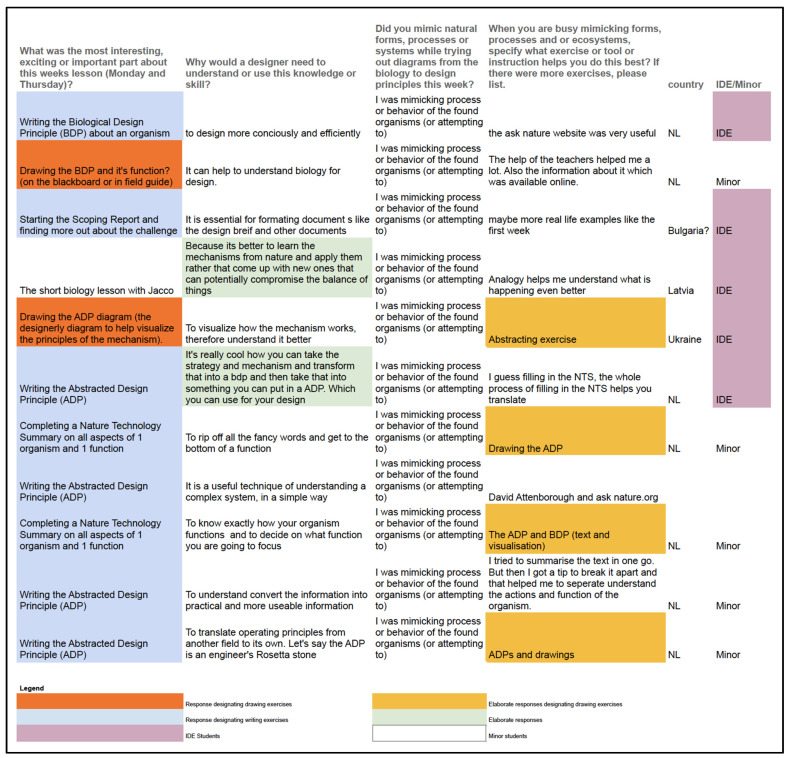
Interview responses of the student cohort mimicking *process* in their designs.

**Figure 11:**
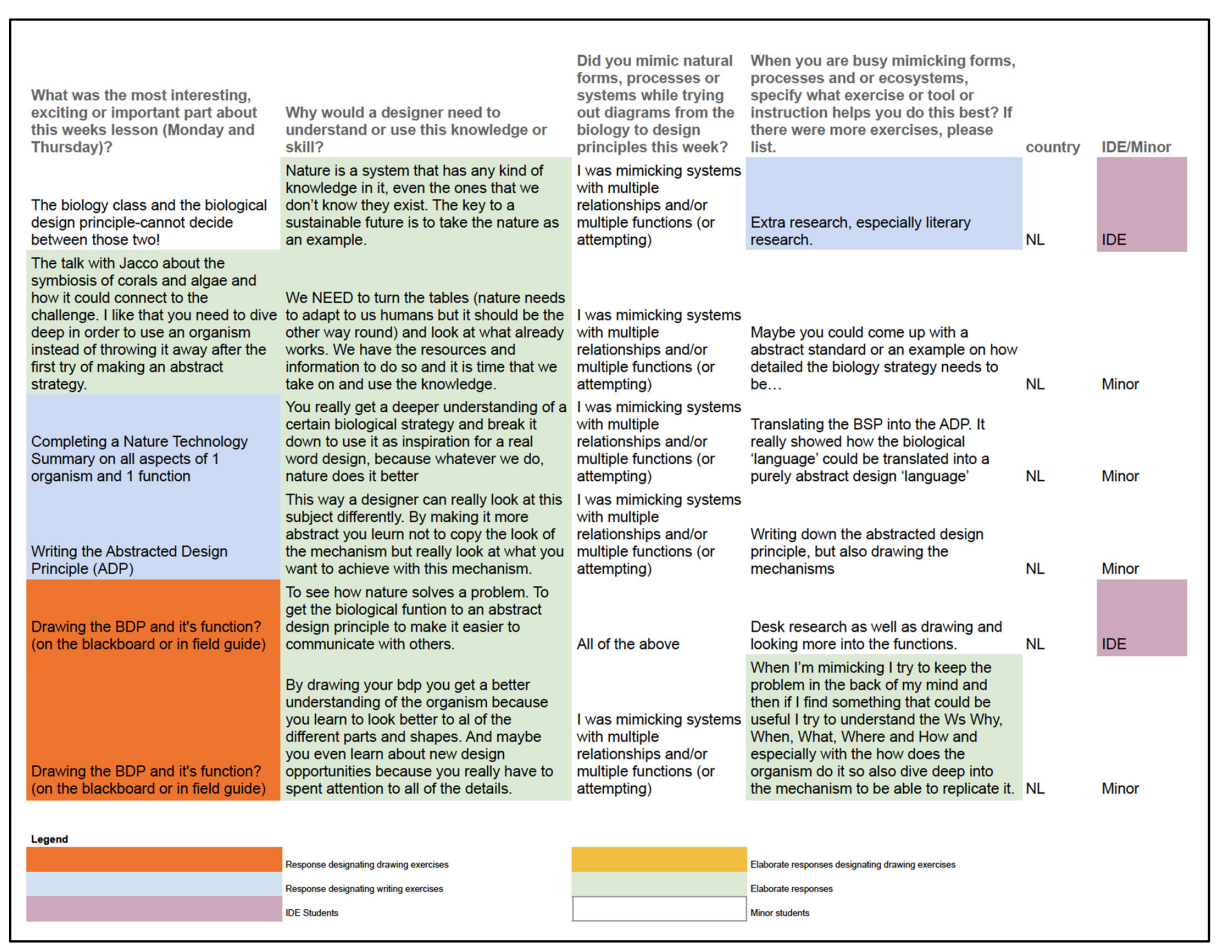
Interview responses of the student cohort mimicking *systems* in their designs.

In this manner, three patterns were found:

(1) Six out of eight students who mimicked form stated that a required drawing exercise of the mechanics involved in the biology or design principles (BDP and ADP), was the most interesting, exciting or important (Figure 9). Self-visualization was thus deemed most important for mimicking forms in the student responses.

(2) Eight of the eleven students who mimicked the process stated that a written exercise (writing the ADP or NTS) was most interesting, exciting or important. (Figure 10);

(3) All who mimicked systems (six students) responded elaborately (Figure 11). The responses on importance of lesson material were evenly split between writing, drawing and “other” exercises in these six responses. All students responding that they felt they were mimicking systems more often chose to describe in more detail why a designer would need this skill than students mimicking form or process.

There were no apparent differences between the responses of IDE students and minor students. All minor students are from the Netherlands, while IDE students are mixed between many countries. There was no striking evidence to support that minor students were more prone to attempt to mimic systems even though there were more minor students responding in this section.

### 3.2. Phase 2 Semi-Structured Interviews of 2022 Cohort

After semester completion, semi-structured interviews were conducted with 10 volunteers from the same 2022 cohort of students, half being IDE students and half minor students. These students were chosen based on their wish to help with the research regardless of how they responded to the former survey (on mimicking form, process or system). A period of 14 weeks between the survey and interviews means they had now reiterated most of their learnings at least once again. The aim of these interviews was to learn specifically which exercises gave them a positive learning experience, which biomimicry exercises had been internalized and what educational factors did they perceive had helped them progress towards systems thinking within the project (if applicable). We also asked what they struggled with during their design process. The interviews took place after the final presentations of their design solutions, 10 weeks after the survey. The open questions were to clarify which factors or elements in the lessons were still fresh in their minds after 10 weeks of applying the tools they had learned. We also wanted to discover what they eventually did with the tools to adapt these to their own challenge of learning biomimicry. Students were invited for a face-to-face reflection session or to join an online Teams discussion where these questions were posed.

We asked:

- What factors helped you reach the level of biomimicry you ended up with?
- What factors in the classroom helped you move forward with this?
- What tools did you use or adapt?
- What did you miss or what would you have liked to repeat?
- What tools do you suggest to enhance ecosystems thinking?

In Table 2, a sample of the interview responses was collected and color-coded to indicate terms that students indicated were helpful in moving them forward with systems thinking, or to indicate a missing element or suggestion. As earlier determined, the discussions during student responses of the semi-structured interviews allowed the researcher to ask multiple times if the response was correctly interpreted. For example, the yellow-marked key words indicate the exact tool mentioned by the student as being helpful for reaching their level of translation from the biological to design mechanism. Their body-language indicated their enthusiasm and excitement during responses. Orange marks the words which demonstrate interest and intent. Green marks indicate tools that, outside of the exercises, showed emotion or direct influence, and blue marks indicate tools that students adapted to continue their learning. Purple marks indicate student suggestions to improve understanding of biomimicry design thinking within the course.

Another example is that when asked what was important to move forward, students responded that the process of *discovering* biological strategies, *using real and relevant challenges*, and *quick feedback loops* were important factors for their learning and a major help for them to move forward quickly and stay engaged. Regarding specifically developing their understanding of systems analogies, teams who wrote multiple Nature Technology Summaries (NTSs) on multiple organisms within a single ecosystem simultaneously helped these teams to discover how the organisms (or stakeholders) are connected and what the underlying relationships can mean. This was not a given exercise, but one that several teams did on their own. We also asked students what could be improved, what was missing or what could help them understand systems better. Multiple students suggested the following:

- Add an explanation of design thinking for non-designers;
- Add an extra motivation boost or exercise before starting the NTS;
- Add a simpler NTS in the first round, before the more developed NTS;
- Add system mapping of relationships and functions of organisms in one ecosystem;
- Add more information on materialization and prototyping.

Patterns that are recognized from the interview responses show that eight out of ten students mentioned the biological design principle and the abstracted design principle exercises as the most helpful in their analogy learning process. Teacher enthusiasm together with quick responses and the fact that the challenges they work on are real and relevant environmental challenges, allowing moments of discovery, are mentioned as factors that cultivate their curiosity to keep learning. It is worth mentioning that the BDP, ADP and NTS exercises were not mandatory to pass the course. Students were asked to complete the exercises if they wanted to learn the biomimicry tools but told that these would not be part of their grade. Tools that were voluntarily adapted or combined in order to learn more about the organism, the workings of a system and to help them evaluate and choose the idea that might have the most impact are the following:

(1) Mapping out the relationships with other organisms in the same ecosystem parallel to the human design challenge;
(2) The Life’s Principles evaluation system combined with the Design Thinking Harris Profile evaluation system;
(3) The combination of additional or multiple biological and abstracted design principles when the direction changed for their stakeholder;
(4) Combining the solutions of teams together to learn more about the relationships between them in one system;

Students also had multiple suggestions to improve the biomimicry design thinking course “Design with Nature”. While IDE students had a background in design, the methodology was completely new for the minor students. One of the most shocking discoveries for our instruction was that we had overlooked this aspect. The suggestion to insert a mini-design thinking lesson for non-design students at the beginning of the semester is a major but welcome addition. As in earlier research, the translation between the biology (BDP) and the abstraction (ADP) was named as a struggle. Suggestions for more time and more detailed instructions before starting were mentioned by almost all students. One student requested more on the cause and effect within a system using causal loops and another wanted to know more about how to continue with biomimicry after the semester was over.

## 4. Discussion

The goal of this study was to measure what the students believed helped them the most and what they considered most limiting. What the students found most interesting or important in the course might not be what they learned the most. It does however show what captures their interest while learning new knowledge and what encourages them to continue “above and beyond”. This could further inform the pedagogy of how to make important topics more interesting to them. By conducting surveys near the start and interviews at the end of each semester, this study examined changes in student perception of their knowledge internalization over a longer period. An apparent difference in student positivity or negativity was not found. The authors agree with Van Ha and Murray (2021) [56] with the statement that regular and immediate oral corrective feedback is considered positive and effective when issued by an experienced teacher and can thus support the accuracy and fluency of the work.

During this study, it is important to note that a single semester on biomimicry design thinking can never match an entire Master of Science on the subject. However, the most essential elements or tools they are provided allow students to understand the basics and to pick up the methodology within their own field of education. By including students in this study as researchers, teachers’ assistants, participants and even as a co-author, we noticed the side-effect that each of those involved was more inclined to contemplate how and why each step was necessary, as compared to earlier cohorts.

Research-based design education, the final design solution (including proof of the design functionality) as well as the manner of assessing students during a semester mix as prioritized elements that an instructor/researcher needs to keep in mind when teaching biomimicry at a university. The authors believe that by addressing these educational elements separately, we highlight the advancements in each, but miss the combined knowledge when taken together as a whole. A series of articles or book chapters that are set up as building blocks might allow for proper definitions and explanation of each, while also allowing one to focus on one element at a time. In the future, we shall look to the work of Yen and colleagues for relevant starting points in our curricular design. The authors acknowledge that there is a possibility that impact bias could have influenced the survey and interview responses. This bias is described as student overestimation of how positive or negative their feelings are about a specific experience (Grimes et al., 2017) [57]. The authors also recognize bias through the influence of enthusiastic and motivated instructors and teachers’ assistants (TAs) on current students. The TAs facilitated the communication amongst the teams of 2022 by their presence and by encouraging the students to ask regular questions for feedback (the TAs, who were former DwN students, could easily associate with their process of design and exploration and share their own experiences and actions). Instructors are influenced by the feedback from their students (Pambookian, 1974) [58], making it plausible that students are affected in the same manner.

### 4.1. RQ 1: Which Elements of Biomimicry Methodology Supported Designers and Non-Designers within a Team to Continue and Increase Their Interest in Learning and Which Factors were Relevant for Specifically Form, Process and System Analogies?

Before the survey, students had participated in the following exercises: Systems mapping, BioBrainstorm, biological design principle, abstract design principle, and the Nature Technology Summary. Two Life’s Principles lessons were also given during this time, but are not examined in this paper, as this was studied and described in depth in an earlier study on systems addressing only this exercise.

From earlier research (Yen et al., 2011; Stevens, 2021) [11,31], we had known the powerful and positive influence for students when discovering biological organisms and translating found strategies to abstract design principles. This positive finding was reiterated in the survey responses. In addition, specific information was obtained about which parts of these exercises, namely, the requirement to draw these themselves, were helpful. However, the translation between the context of biology to that of design remained difficult. In this phase of research, we took apart the physical elements within the exercise to see if there was a correlation between the factors within the exercises that might help students learn as well as progress from the level of form and behavior analogies to that of systems. The authors agree with Baghban (2007) [59], who wrote about the actions children took when creating, drawing and labeling pictures to comprehend the meaning of each process.

We found that drawing and visualization of the biology and the diagrams of abstracted design principles within this biology was deemed important by most of the students practicing form analogies, as was the case in earlier research (Quillin and Thomas, 2015; Stevens et al., 2022b) [55,60]. Students who responded that they mimicked the process or behavior of an organism mentioned more often that a written element of an exercise was helpful.

In the Design with Nature 2022 cohort, more students appear to grasp that the complexity of demonstrating multiple functions and relationships is within reach. All students who said they were practicing system analogies responded more in depth. While there was not another pattern found in the survey regarding which kind of exercise helped them to do this the most, we did, however, pinpoint some helpful suggestions from students during the interviews. In the interviews, the mixing and matching of different organism ADPs to understand the system was mentioned, as well as simultaneously addressing the matching functions and relationships directly related in the design project.

### 4.2. RQ 2: According to Participating Students, Which Exercises within the Phases of Biomimicry Design Thinking Assisted Their Attempts to Apply Systems Thinking?

One pattern that arose in student responses was the visible and extensive manner of writing exercises of those students who had responded to being able to mimic systems, as opposed to student responses of those who replied that they had mimicked form and process (structure or behavior). However, these students did not add extra comments to the multiple-choice answers they could choose from. More research is necessary to see if the learning style pattern will be reproduced in a following cohort, and if specific drawing and writing or listening learning continues to have a more profound effect. New findings from this study are the two exercises we can develop further. The first concerns the combination of ADPs in the ideation phase in a morphological chart to come up with multifunctional ideas, correlating with Smith (2007) [61] on how this tool represents a large qualitative design space. The second finding is to research and combine the ADPs from organisms in a single ecosystem and matching these with the stakeholders, as this appeared to help students generalize and abstract the multiple functions and mechanisms. Doing multiple NTSs on organisms within a single ecosystem helped these students to discover how the organisms are connected (or stakeholders) suggesting that a better understanding of these multiple functions encourages better systems thinking. Both directions seemed to enhance students’ ability to understand the system, and are therefore worthy of further research. Here the authors agree with Vattam, Wiltgen, Helms, Goel, and Yen (2011) [27], where learning how to translate natural analogies into design solutions, and fitting these within a system, remains difficult. If students are encouraged by their own findings, their actions to learn these interconnected relationships are likely to increase the contribution to the sustainability of the final solution. This is, in fact, our goal as instructors of biomimicry: that student solutions will indeed fit within a functioning system.

**Figure 12:**
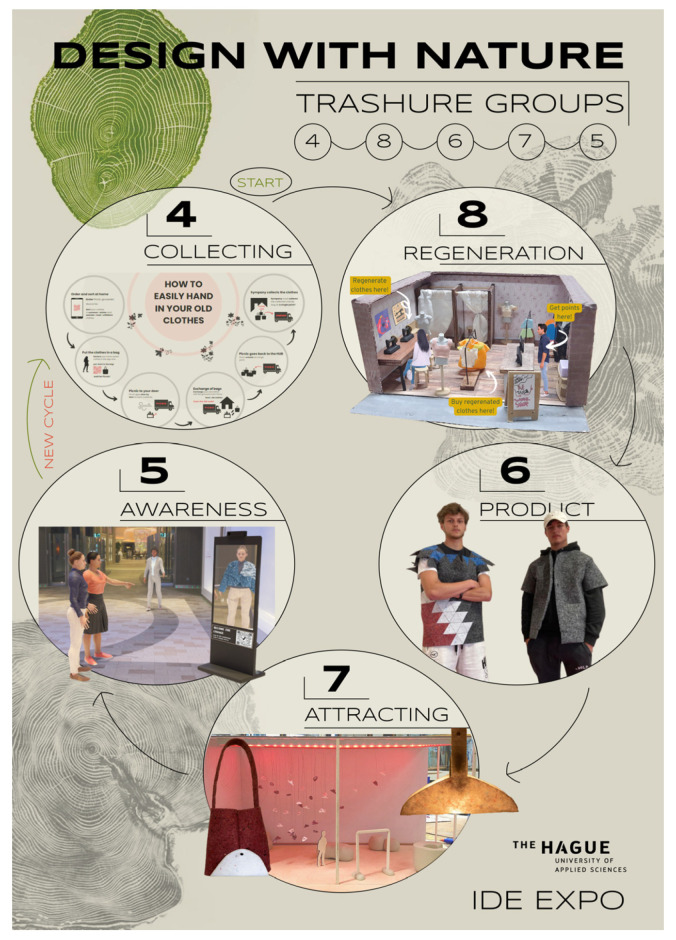
All Trashure DwN Expo poster Circular Textiles, Expo- M. Becker.

At the end of the semester, student teams made a poster of where they fit within the client ecosystem. Teams who had worked with the same main client on the “Circular Textile” project, for example, demonstrated in their posters and presentations how their solutions fit into the stakeholder’s relationships and connections flowing within this particular textile (eco)system (Figure 12). Team 4 focused on the challenge to ease textile collection and sorting methods. Team 8 found methods to regenerate and give new life to old clothing. Team 6 designed new haute couture with the materials deemed “unusable” by the stakeholder community. Team 6 found new ways to attract customers for the new designs and Team 5 concentrated on raising awareness in the currently wasteful and pollutive textile industry, suggesting that collection of thrown-away textiles continues the cycle.

### 4.3. RQ 3: What Pedagogical or Didactical Factors in Biomimicry Methodology Did Students Struggle with or Find Missing?

In an earlier phase of biomimicry education research with educators, Fehler, Bidwell and Singhal, and Stevens (2022a) [55] noted that frequent feedback loops were one of the essential elements of creating reliable abstract design principles (ADPs) within the Nature Technology Summaries (NTSs). Therefore, there was extra attention on quick feedback loops when students worked on the biological design principles (BDPs) and ADPs. The student surveys mentioned in this study support such feedback loops in the learning process, together with the student responses and teacher actions as a result of these responses. Quick feedback loops were set up in the form of frequent peer reviews, cross analysis and regular surveys. This direct response shows that this rapid series of feedback loops likely made a difference during this phase and is hereby reaffirmed as a successful pedagogical strategy for learning (in both the translation phases as well as general learning phases).

From the interviews, we learned that designers and non-designers experienced the start of the course differently. This is liable to be an under-appreciated problem, as many courses in bioinspired design are interdisciplinary in nature and integrate both scientists and designers. We learned that the non-designers first struggled with the combination of the unknown methodology of “Design Thinking” in addition to the new “Biomimicry Design Thinking”. Design students had experience with the first, while none of the students had experience with the latter. An introductory explanation, or “basics of” design thinking for non-designers, needs to be developed to close this gap, as will a slower introduction to the difficult BDP to ADP translation step. Interview responses in which students indicated that they “needed more time” were a common recurrence. An introduction to these steps should demonstrate a variety of BDP to ADP translations and not a single, and more technically oriented ADP, as this had intimidated one of the respondents. Finally, students also mentioned a gap during prototyping. The authors recognize a need to study the research of Rovalo and McCardle (2019) [62], who found that a key ability of prototyping abstractions of mechanisms found in nature (prototyping the ADPs) was the prototype’s ability to define the boundaries between experts in design and biology concerning aspects such as materialization and scale, and its ability to visualize design and collaboration issues.

## 5. Conclusions

The authors’ research aimed to discover factors that can aid emerging professionals to initiate, develop and continue systems thinking biomimicry projects as part of their strategy. While doing so, we aim to help students find ways that suit their own learning style best. By involving students and clients in this research and practicing continuous (peer) feedback loops, we have learned how our biomimicry students became more involved, took the idea of systems thinking to heart and found ways that can be passed on to the next cohort. Without this research, changes in systems thinking may have still happened, but we as teachers would have remained unaware of their occurrence. Initiation of systems starts when students first realize that there are three ways of mimicking nature—following the form, process or system—to achieve a function. The initiation becomes more embedded in the students’ minds when they dive deep into the science themselves and see the rewarding potential. While practicing how the organisms within an ecosystem match their stakeholders’ challenges, development of ideas to solve this challenge begin with a first glance at successful organisms. By looking at the cause and effect of their own design decisions, they can understand even more how their decisions have an impact on the entire system. A following research phase intends to dive into this cause-and-effect element of systems thinking. We intend to add a system-mapping exercise to show relationships and functions of organisms in one ecosystem parallel with the human challenge, as this may help facilitate analogical transfer. Pedersen Zari (2017, p. 1) [63] describes ecosystem services as “the benefits that humans derive either directly or indirectly from ecosystems”, with a distinction between the provision of services (such as collecting water), the regulation of services (such as managing temperature) and supporting services (such as the fixation of solar energy). The distinction of systems thinking within these services aims to understand the impact our students have on the entire related system when designing. Raising awareness of this has been deemed more important than specifically measuring “scores” of success. However, more specific measurement and background criteria will be implemented during the following studies.

Students repeatedly mentioned how stimulating it was to have real and relevant design challenges that addressed the UN’s Sustainable Development Goals. They found that the scientific biological discovery was awe inspiring and cultivated their curiosity. These elements coincide with the Naturalis Biodiversity Center’s BIG 5 of Education, namely: awe-inspiring starting points, real challenges, relevant challenges and challenges that stimulate inquisitiveness and require scientifically sound research (Naturalis, 2015) [64]. Their deeper research and understanding into the entire Nature Technology Summary on one or more organisms or ecosystems was “hedonically pleasing” (Perlovsky et al., 2010) [65] and, thus, encouraged them to continue onwards with this level of design, within the realm of the particular ecosystem.

The final products were not measured within this study’s data. We focused on student responses and what they felt helped them to learn. A more extensive test with test groups with defined and measured end products is planned for next year’s cohort of Design with Nature at The Hague University. Further research shall address the patterns found of diving into ecosystems and the mapping out of the inner organism relationships, as well as a review of causal loops to recognize the cause and effect of decisions made during design. Each is related to stakeholders within a project, and to the combination of strategies and mechanisms these organisms’ relationships offer as a holistic multifunctional ideation element within the biomimicry design thinking semesters.

*“Waste is not allowed back into the resource stream.*

*You must change the mindset that food webs are all within the dynamic system.*

*Interchangeable.*

*Can we change the roles of the organisms into roles of the stakeholders within the system?*

*If something is not covered, make a company link that solves that functionality”.*

Marjanne Cuypers, Seawood Materials, Blue City Rotterdam, 2022.

## Figures and Tables

**Table 1:** Research context, participants and other details.

Institution and Location, Program and Course Name:			The Hague University of Applied Sciences (THUAS), The Hague, Netherlands Industrial Design Engineering, Design with Nature (DwN)			
**2022 Cohort**	**Student Type**	No. of Students	Student Experience Level	Student Background	Student Background in Biomimicry (BMY)	Country of Residence
25 students	Industrial Design Engineering (IDE) students	10 students	Second-year undergraduate students	Industrial Design Engineering	Heard of biomimicry, but no in-depth knowledge	From: Netherlands, Finland, Latvia, Bulgaria, Ukraine, Russia (many students with dual nationalities)
	Students from other programs (minor students)	15 students	Third-year undergraduate students from other programs	Third-year students Dutch spoken Industrial Product Design, Built Environment, Urban Studies, Mechanical Engineering, Physics and Arts and Sciences	Heard of biomimicry, but no in-depth knowledge	From: Netherlands

**Table 2:** Student cohort of 2022 interview and discussion responses—Acceptance, application and gaps of tools.

Responses	Factors Most Helpful to Learn/Understand Analogical Level (F, P, S)	Factors Mentioned as Helpful to Continue Learning Further;Key Words Mentioned That Cultivated Curiosity to Learn	Tools Used/Adapted to Keep Learning Systems Thinking	Missing Elements to Help Learn BMY Better (or Needed Repeating)	Suggested Systems Tools
Student 1IDE	Quick BDP to ADP with instant feedback loops from the instructor.	Writing the NTS gave building stones within a system. Discovering biological organisms motivated me to look for more within the ecosystem. By doing so, I learned what it was capable of, but also what danger it was in.	During the discovery phase, map out the relationships with other organisms in their ecosystem.	ADP was first a shock. Need more explanation that it did not necessarily have to be technical.	-Motivation boost to start the NTS, then it went on its own; and something to aid feedback for ADPs and NTSs
Student 2IDE	ADP gave the “spark” the solution.	Teacher being open and enthusiastic and approachable. Constant feedback. BioBrainstorm discovery of new organisms.	LP Audit and Harris Profile added weight/importance to choose most successful concept.	More time to get even more feedback.	NTS, start with the organism and its function. Then map out what it is influencing in its system. with causal loops
Student 3 MINOR	Going from BDP to ADP.	Talk with the expert (chemist). Finding out how multifunctional everything in nature is. Understanding how useful this is for the Built Environment study with the climate crisis and all.	None.	More time and more help with design process and BDP and ADP for non-designers.	Something to help understand the BDP to ADP template. Or spread it out more to the other units. Or divide the template.
Student 4MINOR	Diving into the biology and chemistry of organisms [BDP].	Being able to continue learning biomimicry in a master’s or an internship (seeing usefulness of biomimicry). Relevancy of the field.	No adaptations to my tools, but did more diving into the chemistry in nature.	More time to research. More design process	Perhaps a planning tool.
Student 5MINOR	Amazing to tackle a real environmental problem-capturing carbon and discovering our product might work.	Good teamwork, accepting the different backgrounds and disciplines and becoming friends during the semester. Real and relevant challenges	We did less biomimicry and more bioutilization, but the discovery part helped us stay enthusiastic to keep learning.	We had to do more research at the end of the project all over again.	-More research;-Knowledge on how to continue with biomimicry after course.
Student 6IDE	Life’s Principles SDGs, NTSs ADPs and BioDesign workshops.	Life’s Principles helped to analyze and be inspired by the deep patterns.	Added BDPs and ADPs after learning how when we changed direction.	How materials could play a important role.	None.
Student 7IDE	Comparing different ADPs, creating overall conclusion/recognition of pattern.	Teacher enthusiasm and direct feedback stimulated us to improve, these were our driving force. We combined “2 functions”.	We connected 2 different ADPs to create a new circular design world.	None.	Add a warm-up before ADP. Discussion on nature, different topics, and link these to nature.
Student 8MINOR	Learning BDPs and ADPs.	I used to only copy shapes and forms from nature but now we integrated function, strategy and mechanisms into our design.	Joined teams to combine designs in 1 system.	Lots of prototypes More design process	None.
Student 9MINOR	Learning BDPs and ADPs.	Some teachers were afraid of me taking this minor, because they thought I was just going to design a beehive [mimic form], but we designed a solution via how sea salps filter their food [process].	Combined biomimicry with bioutilization.	Oral assessments with the team More design process	Rather an oral assessment each unit than a written reflection.
Student 10IDE	The middlemen (between creators and consumers) are like trees that feed others and create value.	Understanding the added value helped us move forward. System map: Combining ADP/organisms in system.	Application of feedback loops to stimulate growth of information and its exchange.	None.	None.
**Patterns**	**8 out of 10 BDP to ADP/NTS** **Value of discovery**	**Value/relevance/real challenges/combi organisms in system** **Teacher enthusiasm and feedback loops**	**LP tool with DT tools** **Combine ADPs**	**More time** **Material knowledge**	**Softer intro to ADPs** **Combine ADP in morph chart**

## Data Availability

All the raw data are stored by the participating university on a Google drive behind a two-factor authentication wall.
